# Internet Access and Use Among Dementia Carers and the People They Support in Australia: Cross-Sectional Survey

**DOI:** 10.2196/68333

**Published:** 2025-08-22

**Authors:** Emilie Cameron, Elise Mansfield, Ama Ampofo, Andrea Coda, Allison Boyes

**Affiliations:** 1 Health Behaviour Research Collaborative, School of Medicine and Public Health, College of Health, Medicine and Wellbeing, University of Newcastle, Callaghan, NSW, 2308, Australia, 61 40420701; 2 Equity in Health and Wellbeing Research Program, Hunter Medical Research Institute, New Lambton Heights, Australia; 3 School of Health Sciences, College of Health, Medicine and Wellbeing, University of Newcastle, Ourimbah, Australia

**Keywords:** Alzheimer disease, caregiver, support person, technology, online tools

## Abstract

**Background:**

Dementia is a major public health priority due to its increasing prevalence and the considerable burden it places on individuals living with the condition and their carers. Internet-based tools can help carers and the people they support to manage daily tasks, access resources and support, track health data, and connect with health care professionals. However, the benefits of these tools will only be realized if the intended users have access to the internet and are confident in its use.

**Objective:**

This study aimed to examine the patterns of internet access and use among dementia carers in Australia and the people they support.

**Methods:**

A cross-sectional survey of carers providing informal support to a person diagnosed with dementia and living in the community was conducted. Carers were recruited through geriatric clinics, aged care providers, and community organizations between July 2018 and June 2020. Consenting carers self-completed a multitopic survey that included study-specific questions about their access to the internet, confidence using the internet, and whether the person with dementia they support was able to use the internet. Multivariate regression analysis identified sociodemographic factors associated with carers' internet access.

**Results:**

A total of 172 carers (consent rate 47%) with an average age of 71.8 (SD 10.91) years completed the survey. Most carers (126/155, 81%) had access to the internet; however, 31% (47/153) reported they were not at all confident in using it. The likelihood of carers having access to the internet decreased with carers’ age (OR [odds ratio] 0.87, 95% CI 0.80‐0.96; *P*=.003). Male carers were 4 times more likely to have internet access than female carers (OR 4.32, 95% CI 1.00‐18.6; *P*=.05). Similarly, carers with private health insurance (an indicator of individual socioeconomic status) were 8 times more likely to have internet access than those without private health insurance (OR 8.45, 95% CI 2.74‐26; *P*<.001). Only 17% (29/166) of carers perceived that the person with dementia they support was able to use the internet independently or with assistance.

**Conclusions:**

Despite high rates of internet access among carers, significant age, gender, and socioeconomic disparities were found, and a notable proportion lacked confidence in its use. Internet use among people with dementia was limited. The digital disparities identified in this study suggest that targeted training to build digital literacy to effectively use internet resources and co-design new technologies is needed. The findings further highlight that in this digital era, alternative methods to deliver dementia care and resources are essential to accommodate those who are unable to access or are less comfortable using the internet.

## Introduction

Dementia is a major public health concern worldwide, contributing significantly to disability, dependency, and reduced quality of life among older adults. The term dementia refers to a group of symptoms that affect memory and other cognitive functions, mood, behavior, and the ability to perform activities of daily living. Alzheimer disease is the most common form of dementia [1]. As of 2022, an estimated 56 million people worldwide were living with dementia, a figure projected to rise to 78 million by 2030 due to population aging, with global care costs set to exceed US $2.8 trillion [2]. People living with dementia often have complex physical, psychosocial, and practical needs. The progressive nature of dementia can lead to loss of independence, increased risk of hospitalization, and social isolation [3,4]. Informal caregivers, often family members, play a crucial role in supporting the person with dementia. However, caregivers frequently report difficulties in accessing clear information, appropriate support services, and respite care. Many experience high levels of stress, anxiety, depression, and social isolation, along with uncertainties about how to manage the behavioral and medical challenges associated with dementia [5-9].

Recent years have seen a seismic shift toward digital health technology as part of the delivery of health care and care services for older adults, including services provided to people with dementia and their carers [10]. These include telehealth consultations, online access to medical records and information, wearable health tracking devices, and web-based peer support platforms. For both people with dementia and their carers, such tools offer promising opportunities to improve care, reduce burden, and promote autonomy [11,12]. Digital solutions confer significant potential advantages over traditional face-to-face modes of support: they allow access to integrated care and support in a timely manner, which may help to de-escalate behavioral symptoms and prevent crisis situations, allow for greater accessibility of support for those living in rural or remote areas, can be accessed at a time and place that suits the user, can be tailored to individual needs and preferences, are easily modified to take into account visual or hearing impairments (eg, option to present information as video or text, read-aloud features), and are likely to be more affordable at a time of increased financial strain [12-15]. In recognition of the multitude of potential benefits of digital technologies, international action plans such as the United Nations Decade of Healthy Ageing: Plan of Action (2021‐2030) advocate for the use of digital technologies to expand access to quality care for older adults [16].

Evidence from intervention studies supports the effectiveness of internet-based programs in improving outcomes for carers, including reduced depressive symptoms, lower perceived stress, increased self-efficacy, and improved confidence in decision-making [17-19]. Similarly, for people with dementia, digital tools have shown potential to enhance well-being and cognitive functioning [20,21]. However, many technologies shown to be effective in controlled research settings have yet to be widely adopted in practice. Reasons provided by older adults for not using the internet include not having a need or interest in it, a lack of confidence, and not having access [22]. Factors such as age, gender, education, income, remoteness, health status including mental health, and digital literacy influence technology use among older adults [23-26]. Reluctance to engage with digital solutions can stem from mistrust, unfamiliarity, or perceived complexity [11,14,27]. Consequently, many carers and people with dementia may be missing out on the benefits of online interventions.

To date, few studies have examined population-level internet use among people with dementia, with existing research largely focusing on specific interventions (eg, cognitive training apps) or usage in early-stage dementia [20,21,27-31]. Studies from various countries suggest rising internet usage among carers [32-34], for example, more than 90% reported internet use for caregiving purposes in Portugal in 2022, but these findings are often based on online convenience samples, which may not reflect broader populations [34]. The extent of carer’s confidence and training needs remains underexplored. This study aimed to address these gaps by exploring how confident carers of people with dementia are in using the internet, the factors associated with having internet access, and whether the person they support is able to use the internet.

## Methods

### Design

A cross-sectional survey of carers of people with dementia was conducted.

### Participants

Eligible carers were aged 18 years and older, self-identified as able to read and write English, and were the primary source of informal practical and emotional support to a person living in the community who had received a confirmed diagnosis of dementia from a medical professional.

### Recruitment and Data Collection

Participants were recruited as part of a larger study examining dementia carer needs [5]. Potentially eligible carers were identified through multiple settings in 2 states of Australia (New South Wales and Victoria) including 10 private geriatrician clinics, 3 community-based respite centers for people with dementia, 6 carer support groups, 34 community-based social groups for older people, and an aged care provider. Staff members within these settings reviewed records and provided potential participants with a study information pack including a cover letter, information statement, hard copy of the survey, and reply-paid envelope for the return of the survey directly to the research team. Alternatively, the study was advertised within the setting, and interested carers completed a study eligibility form, which was returned to the research team to mail out the study information pack. The survey could be completed by pen and paper or online if preferred. A reminder including a second copy of the survey was sent after 4 weeks to nonresponders. Data were collected between July 2018 and June 2020.

### Measures

Participants self-reported their sex, age, postcode, highest level of education, private health insurance status, concession card status, presence of health conditions, and relationship to the person with dementia and completed a measure of depression severity (Patient Health Questionnaire-9 [PHQ-9]) [35]. Participants were asked whether they have access to the internet (Yes or No), how confident they are in using the internet (Very confident or Somewhat confident or not at all confident), and whether the person they support would be able to use the internet if they wanted to (Yes on their own or Yes with help or No).

### Analysis

Analyses were conducted in R version 4.3.1 (2023-06-16 ucrt; R Foundation for Statistical Computing). Descriptive statistics are reported as counts and proportions for categorical variables and mean and SD for continuous variables. Postcode was used to calculate an Accessibility/Remoteness Index of Australia Plus (ARIA+) score indicating whether people live in a major city or regionally [36]. Postcode was also used to estimate the degree of socioeconomic disadvantage using the Socio-Economic Index for Areas (SEIFA) Index of Advantage and Disadvantage (2016) [37].

Factors related to internet access were first modeled individually and then all the factors were included in a multivariable logistic regression model. These factors included age, depression score (PHQ-9), sex, ARIA+ score, SEIFA decile, highest level of education, relationship to person with dementia, living arrangements, private health insurance status, and the presence of health conditions.

### Ethical Considerations

The study was approved by the Human Research Ethics Committees of Hunter New England Health (17/05/17/4.07) and the University of Newcastle (H-2018‐0308). Participation in the study was voluntary. Completion and return of the survey was taken as implied consent to participate. All personal information was stored separately from responses collected in the survey and only deidentified data were analyzed. Participants were not compensated for their participation in the study.

## Results

### Sociodemographic Characteristics

A total of 172 carers completed the survey (consent rate 47%). Participant characteristics are shown in Table 1. Most participants were female (124/172, 74%) lived in a major city (118/140, 84%) and had an average age of 71.8 (SD 10.91) years. The mean PHQ-9 score was 5.50 (SD 5.37), which is classified as mild depression.

**Table 1.** Sociodemographic and internet characteristics of study participants (N=172).

Characteristics	Values^a^
Age (years), mean (SD)	71.81 (10.91)
PHQ-9^b^ score, mean (SD)	5.5 (5.37)
Sex, n (%)	
Female	124 (74)
Male	44 (26)
ARIA+ score^c^, n (%)	
Major city	140 (84)
Regional	27 (16)
SEIFA^d^, n (%)	
Most disadvantaged (decile≤4)	102 (61)
Least disadvantaged (decile>4)	65 (39)
Education, n (%)	
High school or below	67 (40)
Vocational training or University	99 (60)
Relationship to person with dementia, n (%)	
Partner	140 (83)
Parent	22 (13)
Other	7 (4.1)
Living arrangement, n (%)	
Live separately to person they support	12 (7.1)
Live with person they support	157 (93)
Private health insurance, n (%)	
No	42 (25)
Yes	126 (75)
Health conditions, n (%)	
None or 1 health condition	93 (56)
>1 health conditions	73 (44)
Internet access, n (%)	
No	29 (19)
Yes	126 (81)
Confidence using internet, n (%)	
Very confident	52 (34)
Somewhat confident	54 (35)
Not at all confident	47 (31)
Person with dementia able to use internet, n (%)	
Yes, on their own	13 (7.8)
Yes, with help	16 (9.6)
No	137 (83)

a Totals do not all add to N=172 due to missing responses.

b PHQ-9: Patient Health Questionnaire-9.

c ARIA+: Accessibility/Remoteness Index of Australia Plus.

d SEIFA: Socio-Economic Index for Areas.

### Internet Access and Use

Most carers (126/155, 81%) indicated they had access to the internet. Overall, 34% (52/153) of carers reported being very confident, and 35% (52/153) reported being somewhat confident using the internet. The majority of people with dementia (114/166, 83%) were reported as not able to use the internet. A further 9.6% (16/166) of people with dementia were reported as being able to use the internet with help, and only 7.8% (13/166) were reported as able to use the internet independently.

### Factors Associated with Carers’ Internet Access

As shown in Table 2, after adjusting for other factors, age, sex, and private health insurance status were significantly associated with internet access among carers of people with dementia. For every 1-year increase in age, there was a 13% reduction in the odds of a carer having internet access (odds ratio [OR] 0.87, 95% CI 0.80‐0.96; *P*=.003). Male carers were 4 times more likely to have internet access than females (OR 4.32, 95% CI 1.00‐18.6; *P*=.05). Similarly, carers with private health insurance were 8 times more likely to have internet access compared to those who did not (OR 8.45; 95% CI 2.74‐26; *P*<.001).

**Table 2.** Sociodemographic factors associated with having internet access among carers of people with dementia (n=155).

Characteristics	Crude			Adjusted		
	OR^a^ (95% CI^b^)		*P* value^c^	OR (95% CI)		*P* value
Age	0.94 (0.89‐0.99)		.01	0.87 (0.80‐0.96)		.003
PHQ-9 score^d^	1.11 (1.01‐1.22)		.05	1.09 (0.96‐1.24)		.20
Sex						
Female	Reference		—^e^	Reference		—
Male	2.27 (0.80‐8.12)		.16	4.32 (1.00‐18.6)		.05
ARIA+ score^f^						
Major city	Reference		—	Reference		—
Regional	0.76 (0.25‐2.87)		.66	0.81 (0.18‐3.69)		.80
SEIFA^g^						
Most disadvantaged	Reference		—	Reference		—
Least disadvantaged	0.76 (0.33‐1.78)		.52	1.21 (0.41‐3.61)		.70
Education						
High school or below	Reference		—	Reference		—
Vocational training or University	1.82 (0.80‐4.14)		.15	1.42 (0.50‐4.04)		.50
Relationship to person with dementia						
Partner	Reference		—	Reference		—
Parent	5.35 (0.69‐41.6)		.11	4.31 (0.16‐119)		.40
Other	1.07 (0.11‐9.97)		.95	0.46 (0.02‐11.2)		.60
Living arrangement						
Live separately	Reference		—	Reference		—
Live with person they support	0.52 (0.03‐3.02)		.55	32.7 (0.58‐1,845)		.09
Private health insurance						
No	Reference		—	Reference		—
Yes	5.06 (2.15‐12.2)		<.001	8.45 (2.74‐26.0)		<.001
Carer’s health conditions						
None or 1 health condition	Reference		—	Reference		—
>1 health conditions	0.72 (0.31‐1.65		.43	1.17 (0.40‐3.43)		.80

a OR: odds ratio

b CI: Confidence Interval

c *P* values <.05 are significant.

d PHQ-9: PHQ-9: Patient Health Questionnaire-9.

e Not applicable.

f ARIA+: Accessibility/Remoteness Index of Australia Plus.

g SEIFA: Socio-Economic Index for Areas.

## Discussion

### Principal Findings

We found that most (126/172, 81%) carers in our study had access to the internet. The proportion was higher than expected given the average age of the carers in our study was 71 (SD 10.91) years. Data from the Australian Bureau of Statistics suggest that in 2018, 62% of people older than the age of 65 years were internet users and this increased to 76% in 2022 [38]. Other studies reporting on internet use among dementia carers have tended to have a younger cohort and often use internet-based recruitment methods [32-34]. Compared to national data, our sample contained more carers who were the partner of the person with dementia, were older, and were residing in major cities [5]. Our findings suggest that it is feasible to leverage the increasing rates of internet access among older people, including dementia carers, to provide dementia care services, such as e-learning modules, virtual support groups, telehealth services, and online care plans.

Consistent with other studies illustrating the digital divide, our cross-sectional survey found that younger carers were more likely to have internet access than older carers [22,24,32,33]. Men were also more likely to have internet access than women. As a majority of carers of people with dementia are women [39], there is a need to further explore reasons for reduced uptake and develop strategies to overcome barriers to uptake among this group. Those with private health insurance were also more likely to have internet access, compared to those without. Having private health insurance is an indicator of individual socioeconomic status [40]. This finding likely reflects the costs involved in accessing digital technologies including initial financial outlay for equipment and ongoing charges for internet connection [11]. These findings suggest that dementia care resources and services must continue to be accessible through a variety of channels, particularly for carers from vulnerable population groups who often face social and economic barriers to digital inclusion.

Part of eHealth literacy theory posits that in order to effectively use internet technology, carers not only need access to the internet but also the self-efficacy to use it [41]. Accordingly, a systematic review showed that interventions designed to increase self-efficacy in the use of eHealth among older adults were associated with increases in their ability to manage health concerns [42]. We found that only one-third of dementia carers feel very confident in using the internet. There is an opportunity to help carers feel more confident in using the internet through successful eHealth literacy training programs [23,24] such as “Be Connected”—an Australian Government initiative to improve digital literacy for older adults [43]. Promoting the use of such programs may assist in improving uptake of and engagement with a range of digital health platforms. In addition, ensuring carers are provided adequate training in the use of new technologies as they are introduced will help them to feel comfortable, less frustrated, and more likely to use them [11]. Future research should consider the factors associated with confidence in using the internet and ways to build digital confidence so that the full potential of internet-based support systems can be realized.

While internet access among carers in our study was high, our findings revealed that only 17% of people with dementia were able to use the internet either independently or with assistance. This proportion is consistent with data from the Australian Bureau of Statistics which identified 12% of people with dementia as internet users in 2018 and 27% in 2022 [38]. While internet use is low among people with dementia, some individuals do engage online, benefiting from social connection, sharing stories, and accessing additional support [27,29,30]. Despite this, research has primarily focused on the development and testing of interventions aimed at carers, with little attention to the benefits and potential for people with dementia themselves or ways carers and people with dementia could use the technology together [25]. A systematic review of 90 studies found that people with dementia often required support from carers to navigate internet technologies [31]. There is a risk that increased use of internet-based solutions will increase the demands placed on carers and reduce the input of the person with dementia into their own care [31,44]. These findings highlight the need to carefully consider who will be using digital technologies and whether their needs are being met [28,30,45]. Co-designing solutions with people with dementia should be a priority to ensure digital interventions are relevant, practical, and aligned with their abilities, preferences, and online behaviors. Their involvement in the design process can lead to more effective and acceptable support tools.

### Limitations and Future Directions

Our sample included carers who were linked with support systems, such as respite services and geriatricians, and may therefore not be representative of the population caring for people with dementia. The sample was older than the general population of carers in Australia and included few people outside major cities and none from remote locations. It also did not include those who could not read and write English. These groups may face further barriers to the use of internet-based innovations. The sample size was small due to the difficulties of recruiting people from vulnerable populations [46]. Although the majority of data were collected prior to the commencement of the COVID-19 pandemic, rates of internet access among carers were comparable with rates of internet access for older Australians from 2022 [38]. While internet access and user confidence are important factors in ensuring uptake and engagement with digital health programs, other potential barriers to internet use among these populations, such as level of eHealth literacy, fears about scams and security of personal data, and affordability of internet access could be explored in future studies.

### Conclusions

Internet use among carers was found to be high, suggesting that digital health interventions are increasingly feasible for this population. However, disparities in access and confidence remain, particularly among older, female, and socioeconomically disadvantaged carers. Internet use among people with dementia, however, was limited. The findings reinforce the importance of providing dementia care through diverse modalities. To maximize the benefits of digital health technologies, future efforts should prioritize equity in access, build digital literacy through targeted training, and ensure that online support systems are co-designed to ensure that they meet the needs of both carers and people with dementia.

## Abbreviations

ARIA+: Accessibility/Remoteness Index of Australia Plus
CI: Confidence Interval
OR: odds ratio
PHQ-9: Patient Health Questionnaire-9
SD: Standard deviation
SEIFA: Socio-Economic Index for Areas
